# SEM and TEM for identification of capsular fibrosis and cellular behavior around breast implants – a descriptive analysis

**DOI:** 10.1186/s12860-021-00364-8

**Published:** 2021-05-03

**Authors:** Britta Kuehlmann, Isabel Zucal, Clark Andrew Bonham, Lydia-Marie Joubert, Lukas Prantl

**Affiliations:** 1 Division of Plastic and Reconstructive Surgery, Department of Surgery, Stanford University, Stanford, CA 94305 USA; 2 University Center for Plastic, Reconstructive, Aesthetic and Hand Surgery, University Hospital Regensburg and Caritas Hospital St. Josef, 93053 Regensburg, Germany; 3 Stanford-SLAC Cryo-EM Center, Stanford University, Stanford, CA 94305 USA

**Keywords:** Breast implants, Foreign body response, Capsular fibrosis, Cellular behavior, Scanning electron microscopy, Transmission electron microscopy

## Abstract

**Background:**

Capsular fibrosis (CF) is the most common long-term complication in implant-based breast augmentation. It is well accepted that the foreign body response (FBR) instigates the development of fibrotic disease. Our study aims to compare murine and human samples of CF and describe the cellular and extracellular matrix (ECM) composition using scanning and transmission electron microscopy (SEM and TEM).

**Results:**

Miniature microtextured silicone breast implants were implanted in mice and subsequently harvested at days 15, 30, and 90 post-operation. Isolated human capsules with the most aggravated form of CF (Baker IV) were harvested post-operation. Both were analyzed with SEM and TEM to assess cellular infiltration and ECM structure.

An architectural shift of collagen fiber arrangement from unidirectional to multidirectional was observed at day 90 when compared to days 15 and 30. Fibrosis was observed with an increase of histiocytic infiltration. Moreover, bacterial accumulation was seen around silicone fragments. These findings were common in both murine and human capsules.

**Conclusions:**

This murine model accurately recapitulates CF found in humans and can be utilized for future research on cellular invasion in capsular fibrosis. This descriptive study helps to gain a better understanding of cellular mechanisms involved in the FBR. Increases of ECM and cellularity were observed over time with SEM and TEM analysis.

## Background

Implant-based breast augmentation is the number one plastic surgery procedure performed worldwide, with over 50% of breast augmentation patients chose to subsequently replace their removed breast implants with new ones [1]. Explantation is most often due to capsular fibrosis (CF), a commonplace long-term complication. In fact, studies report a prevalence of CF of 2.8 to 20.4% [2–8]. The degree of severity is clinically classified by the Baker score system which includes four stages [9, 10]. The first stage is the least severe and is characterized by a lack of clinical symptoms, while the fourth stage represents the most severe form of CF characterized by breast deformity, stiffness, and pain. Moreover, the severity grade is histologically classified by Wilflingseder et al.: stage I is a thin and uncontracted capsule, stage II is characterized by constrictive fibrosis and absence of giant cells, stage III displays constrictive fibrosis with accumulation of giant cells, and stage IV is characterized by infiltration of inflammatory cells, foreign body granulomas, and neovascularization [11]. Studies found that macrophages and *Staphylococcus epidermidis* within capsules were often associated with CF, though bacterial colonization is likely a promoting factor rather than the cause of it [12].

The pathogenesis of CF has been widely studied and it is thought that the underlying mechanism is a fibrotic foreign body reaction brought about by inflammatory signaling. TNF-α production has been observed in the progression of CF, where it is expressed primarily in collagen-depositing fibroblasts and macrophages around the implant [13]. Conversely, less severe cases of CF exhibit far fewer bundles of collagen [14]. However, accurate implant and capsular surface analysis of both collagen fiber arrangement and cellular components are scarce. Implant surface texture and volume influence host tissue responses and the development of CF [15, 16]. Atlan et al. classified the surface texture into smooth/nanotexture (80–100 mm^2^), microtexture (100 - 200mm^2^), macrotexture (200 - 300mm^2^), and macrotexture-plus (> 300 mm^2^) when using scanning electron microscopy (SEM) [17].

In this descriptive study, capsules of a microtextured interface were examined using SEM, TEM, and light microscopy. We aimed to analyze the effect of commonly used silicone implants on capsule formation. Human capsules were taken from patients with aggravated CF (Baker IV). We sought to identify a murine model for CF that might be more suitable for the characterization of human CF than current small animal models. Customized, miniature silicone implants for mice were used to more accurately recapitulate human-like CF in a murine setting.

## Results

### SEM analysis

Human capsules were explanted at 5 to 9 years after implantation, while murine capsules were harvested at days 60 and 90 to ensure adequate fibrotic development.

**Fig. 1:**
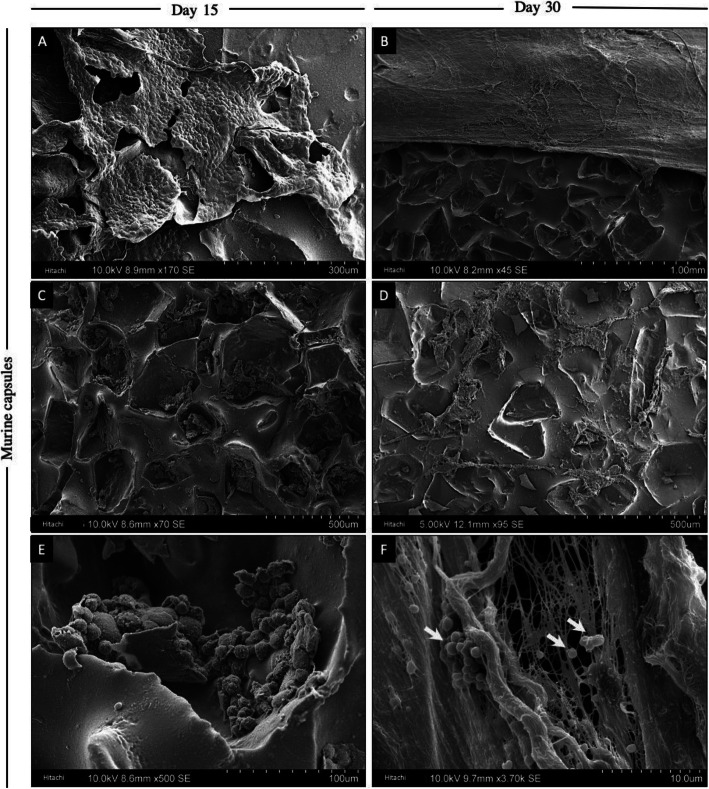
SEM figures of explanted breast implants at day 15 and 30 in mice. **a** and **b** Explanted breast implants with surrounding capsular tissue in murine models are shown at days 15 and 30. **c** and **d** Capsular fragments accumulated in the concavities of the textured implants are shown at days 15 and 30, respectively. **e** White blood cell accumulation (especially macrophages and lymphocytes) in an implant’s pore is presented at day 15. **f** Coccoid bacterial accumulation (white arrows) on a murine capsule of day 30 is shown

Explanted implant surfaces displayed progressive fibrotic accumulation that increased with time Fig. 1a, b. Capsule accumulation was especially notable within the concavities of textured implants (Fig. 1c, e). Interestingly, detached silicone particles and the presence of coccoid bacteria could be found as early as day 30 (Fig. 1d, f). Finally, Fig. 1e shows accumulation of white blood cells in the surface’s concavity. This observation may support the hypothesis that breast implant associated anaplastic large cell lymphoma is associated with textured implants and enhanced T-cell response [18].

**Fig. 2:**
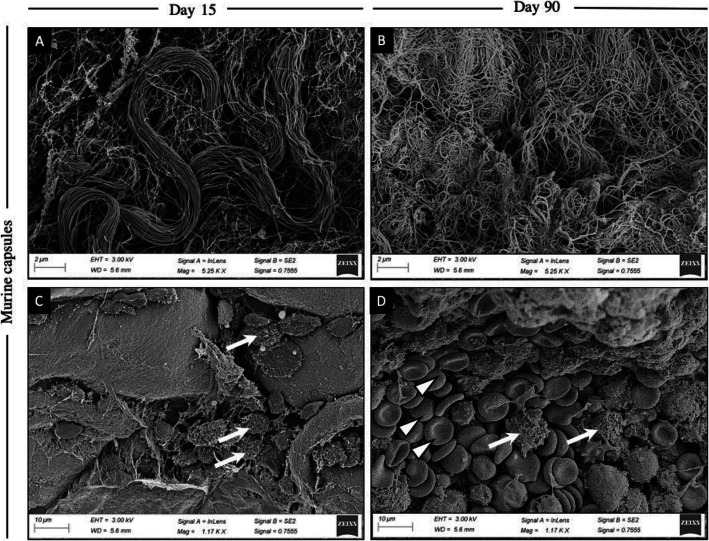
SEM images on collagen arrangement and cellularity at day 15 and 90 in mice. **a** Collagen fiber alignment at day 15 is unidirectional and organized in bundles. **b** At day 90, collagen arrangement appears to be disorganized with loose and multidirectional collagen fibers. **c** Day 15 maintains poor cellularity and is characterized by the presence of white blood cells (white arrows). **d** Cellularity at day 90 shows an increase in erythrocytes (white triangles), whereas white blood cells (white arrows) appear to be reduced in comparison to day 15

Further assessment revealed that murine capsules displayed pervasive fiber-like structures in a similar fashion to that of human samples. SEM images of the murine capsules verified an increase in ECM over time, accompanied by an architectural shift of collagen fibers from unidirectional at days 15 and 30, to a more disorganized fashion by day 90 (Fig. 2a, b). Further, erythrocytes were observed in greater numbers at day 90 when compared to day 15, whereas fewer white blood cells were present at day 90 compared to day 15, suggesting a dissolution of acute inflammation over time (Fig. 2c, d).

**Fig. 3:**
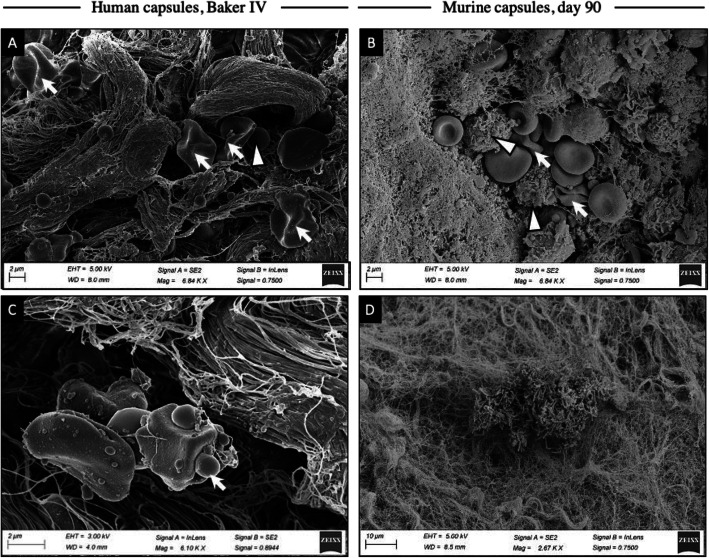
SEM images of human CF (Baker IV) and murine CF at day 60 and 90. **a** Human capsules, Baker IV (human capsules were extracted more than 5 to 9 years after implantation) and **b** murine capsules are shown at days 60 and 90, respectively. Comparable cell infiltration is shown: Both capsules contained coccoid bacteria and a comparable dispersion of blood cells (white blood cells are marked with white triangles), as well as histiocytes (white arrows), that were not found in early stages of CF. **c** The presence of coccoid bacteria (white arrow) around dispersed silicone particles is shown. **d** In one murine capsule, a biofilm-fragment of rod-shaped bacteria is shown in the middle of the multidirectional arranged fibers

SEM images of both murine sections and human capsules revealed a progressive increase in ECM and cellularity within the capsules, with the cellular gradient increasing towards the implant surface at later time points. Cell morphology was comparable in both murine and human models, with the number of histiocytes increasing at later time points in both murine and human capsules, correlating with severe fibrotic deposition (Fig. 3a, b). Furthermore, silicone fragments found in capsules at later stages of fibrosis were characterized by accumulations of coccoid bacteria (Fig. 3c). Interestingly, a biofilm fragment of rod-shaped bacteria was found among multidirectional collagen fibers in a mouse model at day 90 (Fig. 3d).

### Light microscopy

**Fig. 4:**
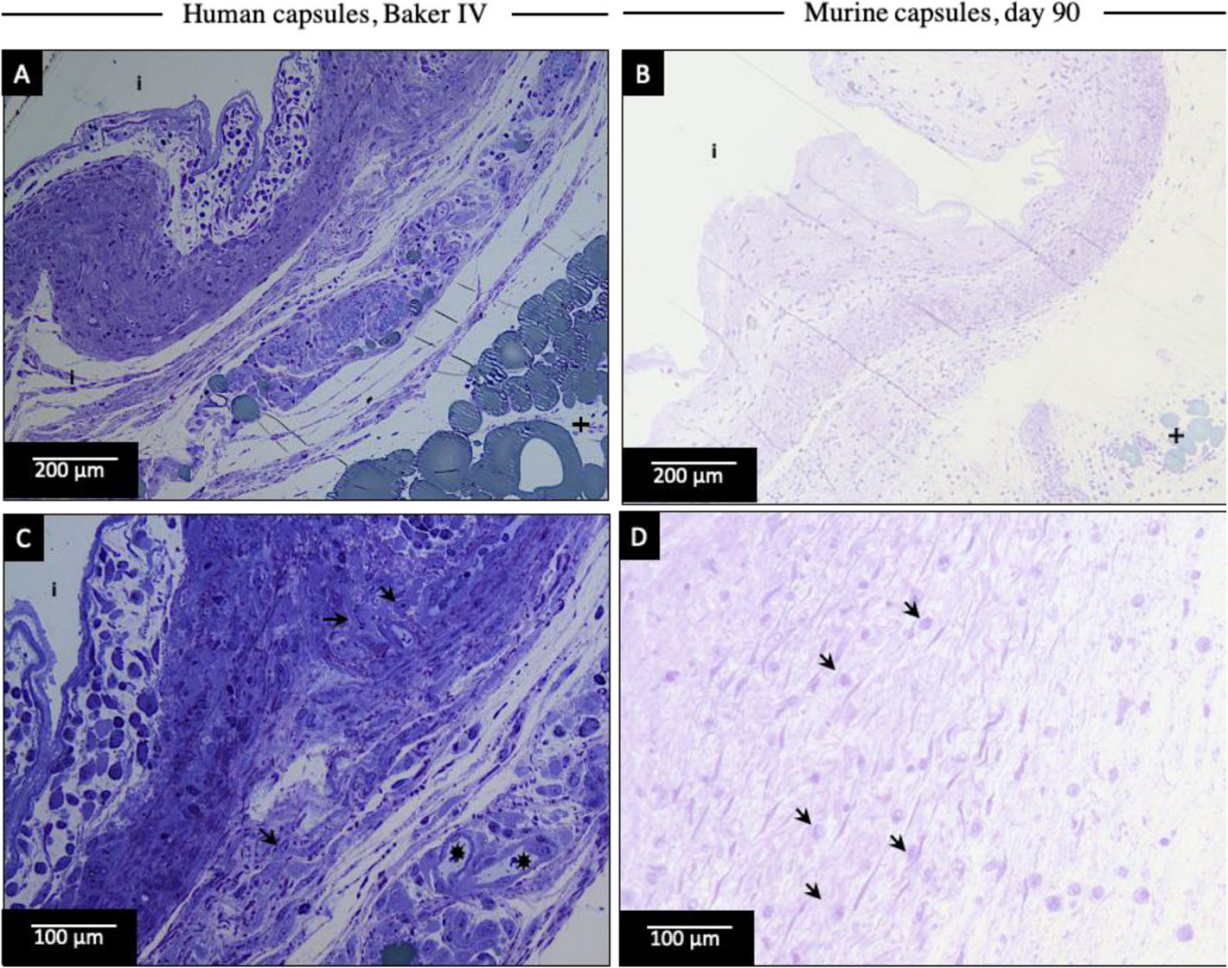
Light microscopy images of human CF (Baker IV) and murine capsules at day 30 and 90. Light microscopy (toluidine blue staining) of human capsular sections, Baker IV (**a** and **c**) are compared to murine capsular sections at day 90 (**b** and **d**). The capsular surface directed to the implant (inner surface) is indicated with (i). Comparable histology was found: The capsules are characterized by collagenous connective tissue building the core of the capsule and the presence of smooth muscle cells with cigar-shaped nuclei, as well as fibroblasts (arrows). Around the outer surface of the capsules, adipose tissue (crosses) and blood vessels (asterisks) can be noticed. Blood vessels are present within the capsule as well (**c**). The capsular layer close to the inner surface is further characterized by lymphocytic infiltration (dark, round nuclei filled with chromatin)

Immediately prior to TEM analysis, light microscopy of human and murine capsular sections was performed. These images displayed a comparable histology of CF in humans, Baker IV and in mice at day 90. Both displayed a collagenous connective tissue-core with several fibroblasts and dispersed smooth muscle cells (Fig. 4). Blood vessels were present in the capsules and the tissue layer at the inner surface of the capsule was characterized by lymphocytic infiltration (Fig. 4).

### TEM analysis

**Fig. 5:**
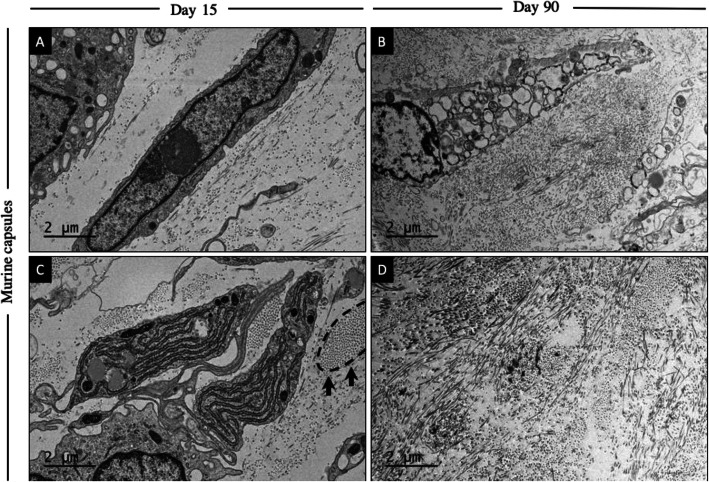
TEM images about collagen fibers in murine capsules at day 15 and 90. TEM photos of murine capsules at day 15 (**a** and **c**) and day 90 (**b** and **d**) are presented. **a** and **c** The collagen fibers around the fibroblast are not as concentrated and appear to be organized in bundles (black dashed line indicated with arrows in (**c**)) at early stages of CF. **b** and **d** As fibrosis goes on, the concentration of collagen fibers seems to increase with a random-pattern arrangement

TEM images of murine sections showed an increase of collagen concentration at day 90 compared to day 15. Furthermore, at day 15, collagen fibers were organized in bundles, whereas at day 90, fibers were multidirectional (Fig. 5). An increase of collagen as an indicator of progressive fibrosis was observed at day 30 compared to day 15 and collagen arrangement was comparable to day 90.

### Immunohistochemistry analysis

**Fig. 6:**
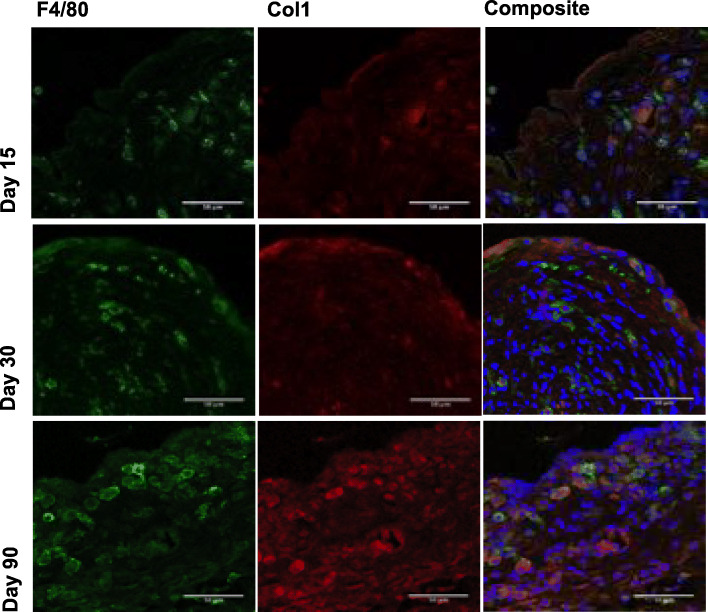
Immunohistochemistry of murine capsules at different timepoints (day 15, day 30 and day 90). 63x magnification. Scale bar = 50 μm. Analysis of immunohistochemical sections confirmed an increase of macrophages and collagen (Col 1) over time. 63x magnification. Scale bar = 50 μm

Immunohistochemistry (IHC) was performed to identify cellularity and ECM deposition in murine capsules at different timepoints (days 15, 30 and 90). IHC confirmed an increase of macrophages and an increase of Collagen deposition (Col 1) over time (Fig. 6).

## Discussion

The implantable device market is rapidly expanding around the world. Since their introduction, the composition of breast implants has been ceaselessly altered in attempts to maximize biocompatibility and reduce complications. In 1992, the FDA restricted the use of silicone implants for cosmetic purposes, but due to insufficient evidence linking such implants to disease, their use in breast augmentation was reapproved in 2006 [19]. CF persists as the most common long-term complication of breast implants regardless of material composition [2–7], bringing about a need for further studies to determine its pathology.

Implant surface texture has been shown to influence the development of CF [11, 20]. In recent years, an emphasis has been placed on developing biomimetic implant surfaces to reduce the foreign body reaction. For example, a novel polydimethysiloxane implant surface was described in a previous study, imitating adipose tissue [21]. As a result, pro-inflammatory genes including ILβ1, TNF-α and IL6 were significantly downregulated. Furthermore, reduced fibroblast and macrophage infiltration was observed in immunocytochemistry and SEM, indicative of a diminished inflammatory response to the foreign body. Here, macrophages were less prevalent compared to original adipose and smooth surfaces. Moreover, fibroblasts were less aligned in the valleys of the surface in modelled adipose surface with secondary texture compared to the original adipose surface with primary texture [21, 22]. Our experiments confirm that larger implantable materials play a crucial role in eliciting a more dramatic and human-like fibrosis in a murine model than those that are smaller, thus making this modified model more suitable than others to study the mechanisms of human CF. Such comparisons between this model and human CF are corroborated by similarities in cellular infiltrate and ECM composition.

In our study, a descriptive analysis of cellularity and collagen arrangement using SEM, light microscopy and TEM was performed. SEM permits topographical visualization and detailed surface examination of solid specimens. Samples are bombarded with electrons that bounce back once they reach their surface, providing in-depth, 3D models [23–25]. Transmission electron microscopy (TEM) is generally used in diagnostic pathology and provides high resolution images (up to 0.2 nm). This allows for the visualization of small intra- and extracellular structures, such as cell organelles, cellular inclusions, microtubules, microfilaments, and intermediate filaments, as well as collagen and amyloids [26].

Microtextured implants were examined, displaying an accumulation of capsular fragments, particularly within the implants’ concavities. Early presence of detached silicone pieces and bacteria at day 30 was observed. The presence of silicone fragments in capsular tissue has been shown to correlate with greater capsular thickness [11]. In our study, we found significant accumulation of coccoid bacteria around these fragments, with rod shaped bacteria found in one specimen. The presence of bacteria in CF has been largely studied in the past, with studies suggesting bacterial colonization could play an important role in the pathogenesis of CF [12, 27, 28] and breast implant associated anaplastic large cell lymphoma (BIA-ALCL) by stimulating an enhanced T-cell response [29–32].

In the cellular analysis, our findings show an increase in histiocytes over time, in agreement with previous studies as an indicator of ongoing fibrosis [33]. According to Siggelkow et al., histiocytic inflammation is more common in patients with clinical symptoms (*p* < 0.001) and around subglandular implants (*p* < 0.096) [34]. In another study, CD68 positive histiocytes were found to be increased in the presence of siliconomas [35].

Histological analysis of the capsular layers in a previous study further suggested the presence of synovial metaplasia within the inner layer, particularly in healthy, uncontracted Baker I capsules, suggesting a protective factor [36]. The middle layer has been observed in line with the capsular border, while the outer layer is loosely arranged [36]. In our histological analysis, we could confirm this type of arrangement in three layers.

Previous studies have identified notable collagen fiber alignment in progressive instances of CF. Highly aligned fibers are found in more contracted capsules, whereas they are loosely arranged and multidirectional in uncontracted capsules [37]. This, however, cannot be confirmed by our findings that show more aligned fibers in days 15 and 30 when compared to day 90, in which the fibers are arranged in a disordered fashion. However, both in vivo and in vitro studies have determined that collagen organization is dependent on the substrate material [38, 39]. TEM analysis showed an increase of collagen fiber-concentration in the ECM at day 90 compared to day 15. Also, previous studies reported enhanced collagen secretion by fibroblasts and myofibroblasts, after acute and chronic inflammation characterized by neutrophils’ and macrophages’ infiltration respectively, dissolved.

## Conclusions

Using this murine model, we were able to confirm the findings of previous studies and further characterize the development of CF at a structural level. When analyzing CF at days 15, 30, and 90, we observed a progressive increase in collagen fibers underlying a structural shift from unidirectional to multidirectional. Further, erythrocytes and histiocytes accumulated over time, suggesting ongoing fibrosis and vascularization of the capsule in advanced stages. Finally, bacterial infiltration was present at all stages in time and was observed to gather around silicone particles and collagen fibers. These findings are relevant as they provide an optimized murine model that allows for better comparison of human CF and may serve as a basis in future studies to develop enhanced biocompatible materials and reduce CF.

## Methods

This experimental study was performed at the Division of Plastic and Reconstructive Surgery, Department of Surgery, Stanford University, USA. Thirty murine and ten human capsules (both of female gender, respectively) around microtextured breast implants were examined with SEM, light microscopy, and TEM at different points in time: day 15, day 30, and day 90 in murine capsules and Baker IV in human capsules. All mice received customized textured, gel filled silicone implants (Polytech Health and Aesthetic, Dieburg, Germany; gel filled, pore size range of 50–900 μm, 2 cm in diameter). Murine implants were placed in a subcutaneous pocket. Human capsules were explanted around microtextured breast implants from an epipectoral pocket. Detailed information about each implants’ manufacturer and generation was not available retrospectively which is a potential limitation of this study. Cell invasion and collagen fiber alignment were assessed.

### Animals

All animals were treated humanely, and protocols used were approved a priori by Institutional Animal Care and Use Committee at Stanford University (IACUC) and Stanford University’s Administrative Panel on Laboratory Animal Care (Protocol No. 28410) according to National Institutes of Health and institutional guidelines. Six-week old female wild-type C57BL/6 mice were obtained from The Jackson Laboratory (Bar Harbor, ME, USA). All mice were housed in sterile micro-insulators. Food and water were provided ad libitum in accordance with institutional guidelines of Stanford University animal care. Ten mice per group were used per each experiment and the experiments were run three times to verify findings.

### Human breast tissue

Human breast capsules were received in FFPE (Formalin Fixed Paraffin Embedded) blocks from the Institute of Pathology, University Hospital Regensburg, Germany. All human capsules examined were formed around microtextured implants to guarantee structural comparison for this study. Approval was given by the local ethic committee in Regensburg (Reference No.: 15–101-0024).

### Scanning electron microscopy (SEM)

For SEM analysis, specimens (breast implants and explanted capsular samples) were rinsed in PBS, fixed overnight in 4% Paraformaldehyde with 2% Glutaraldehyde in 0.1 M Sodium Cacodylate Buffer (pH 7.4), rinsed in the same buffer and post-fixed for 1 h with 1% aqueous OsO_4_. After dehydration in an ascending ethanol series (50, 70, 90, 100% (twice); 5 min each), small tissue (capsule) pieces were critical point dried (CPD) with liquid CO_2_ in a Tousimis Autosamdri-815B apparatus (Tousimis, Rockville, MD), while implants which exceeded the CPD chamber size were treated with HMDS (hexamethyldisilazane) for 30 min (2 × 15 min), before overnight drying in a desiccator. All specimens were mounted onto 15 - 50 mm circular aluminum stubs (Electron Microscopy Sciences, Hatfield, PA), and sputter-coated with 50 Å of gold-palladium using a Denton Desk II Sputter Coater (Denton Vacuum, Moorestown, NJ). Scanning Electron Microscopy (SEM) images captured with either a Hitachi S3400-N Variable Pressure SEM (Hitachi High-Tech, Dallas, TX) operated at 10 kV accelerating voltage and using secondary electron detection or a Zeiss Sigma Field Emission SEM (Carl Zeiss Microscopy, Pleasanton, CA) operated at 3-5 kV accelerating voltage and using InLens Secondary Electron (SE) and Everhard Thornly (SE2) detection. While SEM procedure can cause dehydration artifacts that could negatively impact the tissue processing, histology with respective SEM images of the same sections was performed to ensure that the collagen alignment and hydration state match the fibrotic profile observed in tissue.

### Transmission electron microscopy (TEM)

Samples were fixed in Karnovsky’s fixative: 2% Glutaraldehyde (EMS Cat# 16000) and 4% Formaldehyde (EMS Cat# 15700) in 0.1 M Sodium Cacodylate (EMS Cat# 12300), pH 7.4, for 1 h, chilled, and sent to Stanford’s CSIF on ice. They were then warmed to room temperature (RT) in cold 1% Osmium tetroxide (EMS Cat# 19100) for 1 h while rotating in a hood, washed 3x with ultrafiltered water, then en bloc stained overnight in 1% Uranyl Acetate at 4 °C while rotating. Samples were then dehydrated in a series of ethanol washes for 30 min each at 4 °C beginning at 50, 70, 95% where the samples were then allowed to rise to RT, changed to 100% twice, then Propylene Oxide (PO) was applied for 15 min. Samples were infiltrated with EMbed-812 resin (EMS Cat#14120) mixed 1:2, 1:1, and 2:1 with PO for 2 h each and were subsequently left in 2:1 resin to PO overnight, rotating at RT in a hood. Samples were then placed into EMbed-812 for 2 to 4 h before being placed into molds with labels and fresh resin, oriented, and placed into 65 °C oven overnight.

Sections were taken between 75 and 90 nm, picked up on formvar/Carbon coated slot Cu grids, stained for 30 s in 3.5% Uranyl Acetate/ 50% Acetone, followed by staining in 0.2% Lead Citrate for 3 min. Sections were observed in the JEOL JEM-1400 120 kV with photos being taken with a Gatan Orius 832 4 k X 2.6 k digital camera with 9 μm pixel size.

### Histology

Mice were sacrificed with CO_2_ asphyxiation and cervical dislocation. Murine capsule (tissue) was harvested on day 15, day 30 and day 90 after placing the silicone implant and fixed in 4% paraformaldehyde overnight at 40 °C. The samples were fixed in 4% paraformaldehyde in phosphate buffered solution/saline (PBS) overnight, washed twice with PBS and dehydrated in a 30% sucrose solution for 24 h. Samples were processed routinely and embedded in paraffin. Sections were cut at 1 μm and 3 μm serially for histology staining. Sections were stained with toluidine blue, which has a high affinity for acidic tissue components, such as tissue rich in DNA and RNA. Standardized protocols were used for these stainings with no modifications. Each section was visualized under light microscopy at 5×, 10× and 20× (Leica microscope, Leica DM 4000B; Leica Microsystems, Buffalo Grove, Ill) and photographed using the Leica DFC 500 camera (Leica, Allendale, NJ). Different cell and bacteria types were identified with the help of the aforementioned stain and through their morphology only. Histological sections were examined by a pathologist blinded to the study. Microscopic analyses were performed on ten different fields per slide, using three different slides per sample.

### Immunohistochemistry

For immunohistochemistry paraffin-embedded slides were de-paraffinized and washed twice with 0.25% Triton X-100 diluted in tris buffered saline (TBS) at room temperature for 5 min each. The slides were then blocked with 5% anti-donkey and 5% anti-goat antibodies to reduce non-specific binding of the secondary antibodies for 2 h at room temperature. Primary antibodies (rabbit anti mouse Col 1 (Abcam, ab34710) and rat anti mouse F4/80 (Abcam, ab6640)) diluted 1:500 in TBS with 1% bovine serum albumin (BSA) were added to the sections and allowed to incubate at 40 °C overnight. After incubation, the slides were washed twice with 0.25% Triton X-100 diluted in TBS at room temperature for 2 min each. The secondary antibodies (goat ant-rat AF488 (Thermofisher, A-11006) and (donkey anti rabbit AF594 (Thermofisher, A-21207)) diluted 1:500 with TBS with 1% BSA were added at room temperature for 1 h. During this time the sections were kept in the dark to prevent photo-bleaching. The slides were rinsed twice with TBS at room temperature for 5 min each. Following the secondary stain, the slides were stained with DAPI (DAPI, Biolegend, B222486) diluted 1:1000 in TBS at room temperature for 10 min. The slides were rinsed twice with TBS at room temperature for 5 min each. Aqueous mounting media (Fluoromount G, eBioscience, E099088) was used to mount the tissues. Slides were imaged using an SP8 inverted confocal microscope. ImageJ software (US National Institutes of Health) was used to reconstruct and quantify the confocal images.

## Data Availability

The datasets used and/or analyzed during the current study are available from the corresponding author on reasonable request.

## Abbreviations

BSA: Bovine serum albumin
CF: Capsular fibrosis
Col 1: Collagen 1
CPD: Critical point dried
DAPI: 4′,6-diamidino-2-phenylindole
DNA: Deoxyribonucleic acid
ECM: Extracellular matrix
FBR: Foreign body response
FFPE: Formalin fixed paraffin embedded
HMDS: Hexamethyldisilazane
IHC: Immunohistochemistry
ILβ1: Interleukin 1 beta
IL6: Interleukin 6
M: Molar mass
mm: Millimeter
nm: Nanometer
PBS: Phosphate buffered solution/saline
PO: Propylene oxide
RT: Room temperature
SE: Secondary electron
SEM: Scanning electron microscopy
TBS: Tris buffered saline
TEM: Transmission electron microscopy
TNF-α: Tumor necrosis factor alpha
